# Making water knowledge with Artificial Intelligence: A qualitative study of expert interviews on water diplomacy

**DOI:** 10.1007/s13280-025-02272-z

**Published:** 2025-11-14

**Authors:** Kyungmee Kim, Abeer S. Ahmad

**Affiliations:** 1 Department of Political Science, Swedish Defence University, Stockholm, Sweden; 2 Climate Change and Risk Programme, Stockholm International Peace Research Institute, Stockholm, Sweden; 3 International Institute of Social Studies, Erasmus University Rotterdam, The Hague, The Netherlands; 4 Department of Peace and Conflict Research, Uppsala University, Uppsala, Sweden

**Keywords:** Artificial Intelligence, International water dispute management, Transboundary water management, Water diplomacy, Water knowledge production

## Abstract

Water knowledge, understanding the current and future availability and needs of water, has been critical in negotiating international water disputes. Drawing from expert interviews, this article examines how Artificial Intelligence (AI) tools influence knowledge production and exchange in water diplomacy. The findings suggest that technical strides from AI technologies can enhance data and information objectivity and social learning, potentially benefiting water negotiations and consensus building. However, without addressing political and human challenges, AI tools can exacerbate the risk of eroding trust and spreading dis- and mis-information about politically sensitive water issues. The malicious use of AI poses a serious risk, as negotiators may face increased pressure from public opinion, potentially undermining cooperative progress and escalating tensions over water.

**Supplementary Information:**

The online version contains supplementary material available at 10.1007/s13280-025-02272-z.

## Introduction

Water is a vital resource for human life, functioning societies and ecosystems. Water management is “by definition, conflict management” (Wolf 2009, 67), and the advancement in water conflict management is critical for sustainability and peace (Döring et al. 2024). Over the years, various approaches for water conflict management and prevention have been developed, including water diplomacy. Water diplomacy refers to “political” processes with primary focus on “preventing, mitigating, and resolving” water-related conflicts and tensions over shared water resources by utilizing diplomatic and foreign policy measures (Keskinen et al. 2021, 4–5). With a less technically oriented approach, water diplomacy primarily focuses on political borders rather than the natural and physical borders of international watersheds. Its underpinning is built upon a set of diplomatic principles such as reciprocity, goodwill, and consents. And trust-boosting and norm-sharing have also been recognized as crucial measures to consolidate water diplomacy processes (Grech-Madin et al. 2018; Keskinen et al. 2023). In these processes, water-related knowledge production is a key mechanism of water diplomacy alongside the diplomatic processes that address differing national interests and politics (Keskinen et al. 2021, 7). Knowledge gaps in transboundary water governance seem to be critical concerns in enhancing negotiations and governance (Milman et al. 2020). And further understanding and addressing knowledge making and gaps are critical for ensuring sustainable and peaceful water governance.

This article examines how emerging Artificial Intelligence (AI) shapes knowledge making in water diplomacy by drawing on original empirical materials generated from interviews with practitioners and scholars in the field. New technologies present both challenges and opportunities in managing water resources. The challenges are not limited to substantial water consumption for cooling datacenters for generative AI, and the expanding water footprints of AI is likely to intensify water stress in places where AI datacenters are expanding (Li et al. 2025). AI systems such as generative AI, large language models, and techniques like machine learning, natural language processing, and neural networks have the potential to transform how water data and knowledge are produced, shared, and communicated among water users and between water-sharing countries (Turgul et al. 2024, 281).

Here, *water knowledge* is “not neutral” and is “shaped by the hybridity of water and society” (Usón et al. 2017, 248). This hybridity arises from both hydrological data and a range of social information relevant to water cycle, access and allocation (Linton and Budds 2014). Transboundary water knowledge is shaped by hydrological, socio-economic, and demographic data and information that are relevant for shared water demands, supply, and use, and mutual or contested interpretation of data and information (Milman et al. 2020). Water diplomacy knowledge includes information about negotiating parties, protocols, and an understanding of international norms, all of which contribute to enhanced negotiation skills and diplomatic efforts over transboundary water issues (Grech-Madin et al. 2018). Emerging technologies have played a role in knowledge production in transboundary environmental governance (Bakker 2022). How a set of tools powered by AI can influence conflict management of highly politicized rivers is still to be explored, according to the experts interviewed for this study.

A growing number of initiatives and research works utilize AI technologies, and this calls for a better understanding of knowledge production with AI and its impact on policy. Various AI tools for environmental risk assessment have been developed in recent years by international organizations and national governments. These AI initiatives include the UN Environment Programme’s Strata, the World Food Programme’s HungerMap, the German Federal Foreign Office’s now-finalized PREVIEW program, the Water, Peace, and Security program supported by the Dutch government among others. Additionally, AI-related technologies have been increasingly used in research works on transboundary water governance and transboundary water-induced hazards modeling (ter Horst et al. 2023b; Tran et al. 2024; Motta and Koehler 2025; Palmate 2025). Knowledge produced through these research efforts and initiatives has slowly begun to inform national governments on how to manage shared rivers and better understand the associated risks. For instance, the Government of Vietnam, a downstream country that shares the Red River with its upstream neighbor, China, has increasingly relied on machine learning to predict water levels in upstream reservoirs for disaster response, due to the lack of sufficient hydrological data shared by China (Motta and Koehler 2025, 7).

This article focuses on specific aspects of AI that are relevant to knowledge production and processes of water diplomacy. More broadly an AI system can be defined as “*a machine-based system that, for explicit or implicit objectives, infers, from the input it receives, how to generate outputs such as predictions, content, recommendations, or decisions that can influence physical or virtual environments*” (OECD 2024). Currently, AI systems are fast evolving in terms of objectives, scope, and technologies, and complex systems are emerging that challenges existing governing institutions and state sovereignty (Usman et al. 2023). In the context of water governance, AI systems can be developed for an integrated solution that allows to detect and analyze forecasted hazards and vulnerabilities linked to climate change and future environmental trends (Kim 2025). However, these outputs generated by AI systems are not quite knowledge that is “an iterative process whereby study and experience can shape and then reshape knowledge based on observed evidence” (Dickerson 2022, 737). Rather than informing to a dynamic, reflexive understanding of environmental issues, AI-generated outputs risk reinforcing static or technocratic representations of complex systems. The prospect of machine-generated knowledge and AI systems to dominate climate and environmental governance entails various risks including narrowing the scope of climate governance and supressing environmental activism (Dauvergne 2021). The ambiguity and uncertainty of technology in knowledge production similarly poses challenges in international climate governance (Machen and Nost 2021). The adoption of AI systems in environmental governance would further the process of representation of the environment that is less inclusive while the role of private sector would be emphasized (Kloppenburg et al. 2022). While much attention has been given to the potential risks of AI in broader environmental governance, significantly less focus has been placed on its role in specific domains such as water diplomacy, which is a gap this article seeks to address.

The intersection of AI and water diplomacy remains largely unexplored in existing research, and this article attempts to contribute to this area. First, its main contribution is grounded in empirical data that provides insights into the role of AI in water diplomacy. Additionally, the theoretical framework presents archetypes of knowledge, contributing to a critical debate on water knowledge by deconstructing the assumptions of neutrality and stressing its social hybridity. The research illustrates how AI-generated data and information can transform various aspects of knowledge relevant for water diplomacy. While claims of objective knowledge and the politicization of data may intensify, the potential for social learning through open-source data and tools could be strengthened.

The article is organized as follows. The following section outlines existing perspectives of water diplomacy and the role of knowledge and presents a theoretical framework for further analysis. The methodology section outlines the expert interview method, followed by the result section. The discussion synthesizes the findings and delves into the broader implications of technological innovation and transboundary water governance. The article concludes with policy-relevant insights from the findings.

## A theoretical framework: Knowledge in water diplomacy

Managing water disputes between countries involves complex processes. Uncertainties in data and information, tensions around data and information share, and disputed knowledge complicate dispute management (Al-Saidi and Hefny 2018; Barua and Vij 2018; Hasan et al. 2023). Hydrological, meteorological, ecological, and socio-economic data are critical in constructing claims by countries about how much water is necessary now and for the future, further informing negotiations over equitable and reasonable utilization and the avoidance of significant harm (Mukuyu et al. 2020; Anghileri et al. 2024). Previous research highlights the various roles that data and knowledge plays in international water dispute management. In this section, we provide an overview of different conceptual approaches to knowledge relevant to water diplomacy. Here, it is assumed that knowledge is produced from an iterative processed by studying information and experience (Dickerson 2022). Depending on the assumptions on and aspirations for knowledge generation (Machen and Nost 2021), the nature of knowledge can be characterized as objective, politicized, or socially produced. These perspectives are neither mutually exclusive nor collectively exhaustive, and they share commonalities and differ in ontological and epistemological characters.

Despite varying approaches to the role of knowledge, there is general agreement among scholars and practitioners that political and geopolitical factors fundamentally shape water interactions (Koff et al. 2020; Mirumachi 2020). Water diplomacy as a deliberate political process focuses on influencing this political problem through foreign policy means and diplomatic engagements (Keskinen et al. 2021; Sehring et al. 2022), and this effort also involve enhancing credibility, legitimacy, and saliency of information (Wheeler et al. 2018). While we generally recognize the centrality of power and politics, the following theoretical approaches detail three different roles that knowledge plays.

### Pursuing objectivity in data and knowledge production

The first approach emphasizes the objectivity of knowledge based on a set of positivist assumptions. *Objective knowledge*—if it exists—departs from the perspective that believes the existence of neutral knowledge or truth that can be learned and found through scientific research. It would differ from *credible information* that is grounded in scientifically adequate methods and data. Such perspective has inspired neutral, unbiased, and more accurate data and information can serve as a departure point for mediating tensions (Anghileri et al. 2024). Previous research highlights the value of non-contestable and trusted data in water negotiations (Gerlak et al. 2011; Mukuyu et al. 2020), and a lack of data generates a challenge when parties do not agree on basic facts in prior to negotiations. Scientifically credible and policy relevant information can be served as a useful tool in water diplomacy because such information can help with political conflicts. For instance, Hasan et al. (2023, p.792, italics added) note, “[I]t is not only difficult for riparian states to ignore established and *emerging scientific facts* provided by advances in technology and made available on non-political online platforms.”

Water knowledge regarding hydrological flows in particular and socio-economic data to a degree is highly state-centric, because most hydrological data has been produced by states and in some cases kept in secrecy. When water data sharing is constrained, remote sensing data by a third party can inform and facilitate discussions on how to operate water infrastructure between countries (Thu and Wehn 2016; Hassan et al. 2023; Yalew et al. 2023). More precise measurements and hydrological models can be developed through technological advances, and this can help reduce information uncertainty in transboundary governance (Jeon et al. 2018; Bakker 2022; Morovati et al. 2023; Salhi and Benabdelouahab 2023; Sattar et al. 2024). On one hand, information uncertainty can promote collaboration and benefit advancing negotiations (Bhatia et al. 2017). The lack of reliable and quality data, on the other hand, exacerbates transboundary disaster risks and tensions around water allocation (Thu and Wehn 2016; Mianabadi et al. 2020; Mukuyu et al. 2020).

While reliable data and credible information are crucial for cooperative water governance, an emphasis on neutrality and objectivity of knowledge engenders problems in the attempts to solve political issues with technical approaches. Prioritizing neutrality and objectivity in knowledge production tends to privilege expert knowledge produced by specialists who are seen “neutral authority” in environmental negotiations (Rietig 2014, 152). Such perspective reflects “a positivist worldview that couples the universality of scientific and technical knowledge with the notion that this knowledge is value-free and neutral”, portraying experts as objective actors (Karvonen and Brand 2009, 217). However, the pursuit of objective knowledge may complicate rather than be instrumental to water disputes that overlap with political tensions and uneven power dynamics as Fröhlich (2012, 146) writes, “increasing ‘objective’ data engenders a process of *emotionalization, politicization, and securitization*” leading to “a competition between ‘right’ and ‘wrong’ knowledge, different constructions of reality”. This critique connects closely with the second perspective, emphasizing the inherent political nature of knowledge in international water disputes.

### Politicized and contested knowledge in water interactions

Water diplomacy, as a field focusing on a deliberate political process around water, has contributed to the broadened understanding of political drivers in water negotiations and knowledge generation processes. In various ways, data and information influencing policy decisions are produced through and shaped by political factors: power asymmetry, divergent interests, and equity concerns regarding shared waters (Fröhlich 2012). Even ‘credible’ information with scientific adequacy, gathered through ‘legitimacy’ procedures of inclusive and fair participation, can be contested (Wheeler et al. 2018), if the policy implications of such information are perceived as political risks (Zielinski et al. 2023). The political use of knowledge is typically *imagined* as using scientific research a *weapon* against opposing parties, but this is less likely in multilateral settings (Rietig 2014, 154). Instead, a negotiating party can instrumentalize information and research findings and politically maneuverer the process to its benefit. Knowledge producers such as researchers and experts become aware of the national positions and may adjust their positions accordingly, illustrating a politically influenced knowledge production process (Rietig 2014, 156).

As explained earlier, even if experts with advanced scientific tools may be perceived as *neutral authority* with scientific credibility, it is difficult to avoid politicization of data and information relevant for water diplomacy. This issue is likely to persist with advanced technologies and tools harnessing these technologies. Uncertainties inherent in technical tools for hydrological modeling and scenario analysis reduce their credibility and legitimacy in politically sensitive situations, and the risks of errors and biases cannot be fully mitigated (Yalew et al. 2023). Besides inaccuracy concerns, every socio-hydrological model entails assumptions and aspirations that are subjective and thus contestable (ter Horst et al. 2023a; Wheeler et al. 2023). Ter Horst, Srinivasan et al. (2023) argue that new technical tools may require revisions to existing legal and institutional frameworks concerning data sharing and information exchange. Considering existing challenges in negotiating water agreements, starting a new process concerning the governance of technological tools and information is likely to face challenges, especially for governments that consider water-related data as a matter of national security.

The politicization of water-related data and information can further deteriorate confidence and trust among parties seeking negotiated settlements. Trust is an integral part of international water dispute management. Building trust requires navigating historical, geopolitical, and sociocultural factors that significantly influence cooperative efforts, including knowledge production and sharing (Yasuda et al. 2018; Hasan et al. 2023). Information and data sharing itself can become a subject of contestation and strategic manipulation, especially when certain information seems to undermine national interests (Zielinski et al. 2023). For instance, the downstream impact assessment of the Grand Ethiopian Renaissance Dam on the Nile River has been highly contested due to its significant implications for upstream Ethiopia and downstream countries, Sudan and Egypt. The ongoing debate illustrates how different interpretations of data, key assumptions, and policy preferences shape the understanding of water scarcity related to the controversial dam (Salhi and Benabdelouahab 2023). And this is an area where AI-related technologies are being increasingly explored (Ahmed et al. 2025; Gebremichael et al. 2025).

Rather than contesting the legitimacy and accuracy of knowledge through counter-expertise, establishing common ground through information sharing and knowledge production can positively contribute to advance water diplomacy. Building consensus through repeated interactions and knowledge sharing has been suggested in as part of water diplomacy interventions. The following section describes the approach emphasizing the role of information sharing and knowledge production in consensus-building.

### Learning and producing water knowledge together for trust and consensus building

A shared understanding has been considered as a departure point of consensus building among water-sharing parties to resolve conflicts. This shared understanding can be fostered through activities that exchange information and share technical details and establish *mutually sanctioned knowledge* (Sadoff and Grey 2002). Knowledge co-production is a crucial element in international water dispute management. Common understanding and knowledge allow disputing parties to “listen to other parties and the mediator” (Rajasekaram et al. 2003, 456), which can avoid situations when one party exerts power over another.

Informal dialogues can contribute to establishing a common understanding that incentivizes cooperation by bridging gaps and strengthening collaborative incentives (Mirumachi 2020). The co-production and sharing of knowledge through social and formal interactions have been central to water diplomacy policy making. These activities aim to provide opportunities for mutual learning between parties through social learning, which involves combining various sources and types of information related to water (Pahl-Wostl et al. 2007; Berkes 2009). Enhancing knowledge production and availability holds promise for improving trust and confidence among parties in water disputes (Keskinen et al. 2023; Sharipova 2023).

Reciprocity and goodwill in diplomatic relations influence co-production of knowledge, which contributes to trust-building. Reciprocity is a fundamental principle in diplomacy (Faizullaev 2024), and states’ reciprocal obligations not to harm their water-sharing neighbors are crucial in water negotiations (Zhong et al. 2016). Goodwill in diplomacy involves “the willingness to reach a constructive and mutually acceptable solution that leads to peaceful coexistence” (Faizullaev 2024, 11). In the context of water diplomacy, goodwill extends beyond actions like releasing extra water during droughts and sharing information (Zhang and Zhang 2021) to engage in diplomatic exchanges and negotiations, even if the outcome may be less favorable for maintaining a peaceful relationship (Vu and Nguyen 2024). This is also the strength of water diplomacy, a process that utilizes diplomatic tools and principles to address water issues, fostering constructive engagement over transboundary water challenges.

### Three perspectives on AI’s influence on water diplomacy knowledge

The perspectives outlined in "Pursuing objectivity in data and knowledge production" to "Learning and producing water knowledge together for trust and consensus building" sections form the basis of a theoretical framework to address the intersection of AI and water diplomacy. As discussed earlier, *water diplomacy knowledge* refers to the understanding gained from both socio-hydrological information and insights into negotiation processes, parties, norms, and interests, contributing to the prevention, mitigation, and resolution of transboundary water conflicts. The application of AI-related technologies and the development of AI systems in the field influence how knowledge is produced, shared, and used in water diplomacy processes. The theoretical framework builds on objective, politicized, or socially produced natures of knowledge, which are further elaborated in the following.

Firstly, the neutrality and credibility of data and information relevant to transboundary water disputes can be influenced by the adaptation of AI tools in various stages of water diplomacy processes. By leveraging advanced algorithms and data analytics, AI systems hold promises for more accurate and comprehensive assessments of water resources and their usage. This perception is based on the assumption that AI is less biased and independent from human interpretation and political influence, although biased database, algorithmic bias, and programming biases in AI systems are concerning (Debnath et al. 2023). These risks are particularly challenging to detect and tackle when AI systems lack transparency and are difficult to be explained (Rudin 2019).

Then, the questions about AI’s role on producing and distributing mis-and disinformation about water, deepening political divides and disputes. The very tools that can enhance knowledge accuracy can also be manipulated to distort information for political gain. For example, algorithms may be employed to selectively present data or generate misleading narratives about water scarcity, usage, or contamination. This risk of manipulation and politicization can be reinforced by unequal technical capacities of negotiating parties (Debnath et al. 2023). Power asymmetries among national governments, along with reliance on private companies, contribute to concerns about deepening political conflict (van der Vlist et al. 2024; Borines et al. 2025). These risks flag one of the potentially harmful impacts of AI on water diplomacy knowledge production.

The third proposition focuses on AI's potential to enhance consensus-building through learning and co-production. AI tools can help develop the capacity of weaker parties who may lack the technical or human resources. This equalizing effect of AI can benefit all parties, promoting further progress in building consensus around water use and water infrastructure development. For this equalizing effect to be realized, human monitoring is required to ensure knowledge that benefits less capable party and populations is equally prioritized (Debnath et al. 2023). The following section outlines the expert interview method in detail as the main method for data collection for this research.

## Methodology: Expert interview

Expert interview refers to the targeting and access to ‘expert’ participants as a data collection method (Littig 2009). The method can be justified especially when the research inquiry requires insight into policy processes by individuals who have intimate knowledge. Interviewing experts in the field of water diplomacy is a highly relevant method for examining the propositions, as these experts hold relevant knowledge about specific gaps that AI may help address and challenges it may exacerbate. Interviews of policymakers and experts have been widely used in research into environmental policy processes including in the water sector (e.g. Tawfik 2019; Olive 2020). Water diplomacy is ultimately a policy process or a particular *modus operandi* for managing shared water resources (Keskinen et al. 2021). And the experts with relevant knowledge and experiences can provide their assessments on how technical tools have provided data and information to support political processes and AI systems may contribute to the field in the future. Expert opinions with empirical examples drawn from their experience and knowledge offer insights into the proposed research question.

Semi-structured interviews of 16 experts with a minimum of 10 years of experience include five scholars and 11 practitioners (Table 1). Our sample includes twice as many practitioners as scholars, and as a result, the interviews more prominently reflect practitioners’ insights. The interviewed scholars who hold academic positions are based in Europe (3), North America (1), and Asia (1). Practitioners are based in Europe (4), Asia (3), North America (3), and Africa (1). Two North America-based practitioners focus on Asia and Africa in their respective work. Initially, the authors identified 25 ‘water diplomacy’ experts through the review of key scholarly works and policy documents. Some of these experts have been involved in commissioning studies using advanced modeling including machine learning and exploring the use of AI tools in their research and work. Practitioners include former government officials who were mediators and negotiators, former and current members of River Basin Organizations (RBOs), and experts in think tanks. Not all interviewed experts work closely with a RBO, but most of them have worked with RBOs with relatively high technical and financial capacity (Schmeier 2012). Among 25 experts contacted by email, five declined the request, and four did not reply. The interviews were conducted online during October–November 2023. The timing of the interviews was an important consideration because people’s opinions on AI could be influenced by events and news happening around them.

**Table 1:** A list of Interviews. P: Practitioner; S: Scholar

	Employment	Geographical expertise	Capacity of transboundary basin organizations	Years of experience	Gender	Date
P-1	Government	Europe	High	20–25 years	M	03-Oct-23
P-2	NGO	South Asia	n/a	10–15 years	M	04-Oct-23
S-1	University	Africa	High	10–15 years	M	12-Oct-23
P-3	Former diplomat	Global	n/a	15–20 years	M	13-Oct-23
P-4	Former diplomat	Global, Africa	High	25–30 years	M	16-Oct-23
P-5	River Basin Organization	Southeast Asia	High	10–15 years	M	16-Oct-23
P-6	NGO	Africa	High	30–35 years	M	19-Oct-23
P-7	NGO	Central Asia	Low	10–15 years	F	20-Oct-23
S-2	University	Asia	High	10–15 years	M	24-Oct-23
P-8	NGO	Central Asia	Low	20–25 years	F	25-Oct-23
P-9	Research institute	Middle East, Africa	Medium, high	10–15 years	F	25-Oct-23
S-3	University	Global, Middle East	n/a, medium	30–35 years	M	25-Oct-23
S-4	University	Europe, Southeast Asia	High, high	20–25 years	M	26-Oct-23
P-10	NGO	Asia, Southeast Asia	High, high	15–20 years	M	27-Oct-23
P-11	NGO	Global	n/a	10–15 years	M	30-Oct-23
S-5	University	Global, Southeast Asia	n/a, high	15–20 years	F	10-Nov-23

It is important to acknowledge the selection bias in the interviewee sample, namely less representation of women. 4 women were interviewed out of 16 experts. The initial list of 25 experts includes 10 women, but six of them declined the request with reasons of their lack of AI knowledge and availability. Their ‘lack of knowledge’ was not a reason given by any men as the reason to decline the request. The gender gap in confidence perpetuates in various settings (Carlin et al. 2018), and this may be one reason of the limited participation of women experts for this research. Another reason may be that women have less time for non-essential work due to their professional and personal commitments (Barrett and Barrett 2011). Then, there is a call for greater participation of women in water diplomacy, given their underrepresentation in this field (Sehring et al. 2023).

The interview sample does not equally represent the Global South perspectives. It's also worth noting that most experts—except for four—were based in the Global North, predominantly in Europe and the US. This geographical bias suggests that the empirical data may not fully capture perspectives from the Global South. Consequently, the insights presented in this study may primarily reflect male and Western viewpoints, which poses a limitation on the generalizability of the findings. These sampling biases underscore the pressing need for decolonization within the field of water research.

Expert interviews carry risks of subjectivity and a lack of validation, and such risks can be mitigated by systematic approaches to these problems (Berry 2002). The interview protocol for this research includes a structured questionnaire (Appendix S1). The interview transcripts were inductively coded. First, the main points raised during the interviews were thematically coded using a qualitative text analysis program. These themes were then further analyzed and categorized according to the theoretical framework outlined in "A theoretical framework: Knowledge in water diplomacy" section. The results and discussion in the subsequent sections are based on the following interview quotations (Tables 2, 3, 4, 5). This systematic approach maintains the structure and focus in presenting the data and the theoretically guided analysis.

**Table 2:** Technical strides for objective knowledge making

Interview	Quotation
P-2	If we have access to quality data good for example models which can actually predict how much water will be coming to the system in the next one year, two years, five years or 10 years. How much drains we need to have. How much capacity of water storage we need to have to store that water and use it as an opportunity…I think that the brightest side of artificial intelligence, is that we can actually foresee the future…We can think about the world 100 years later
P-4	[E]specially if we start building automated responses into how governments and parties respond to each other, then things are going to move so fast that I don't think we as human beings are going to be able to keep up with that and be able to think through some of those things
P-5	[R]einvigorating our data system makes sure that it is up to date, accurate, fast, for example, in terms of flood and drought forecasting. This is something to do with life and death of the community who are living downstream of development project hydropower, so this is one part, also the update of our decision support system that are vital to providing scientific evidence or information to decision maker
P-6	[S]o that sharing is a real issue when it comes to transboundary water management. Now with remote sensing people trying to improve things and there is advancement, huge advancement but not yet fully providing all kinds of data sets. I guess that's the kind of core of the disputes about protecting their consumptive use because the downstream countries also are protected by the international law at least to maintain this flow
P-9	[A]lso, the screening and verification process will also require some sort of either human detective work that will take a long time to find something or it also can be done with the automated AI detection for generative text and images
P-10	[W]e [think tank] work along a theory of change where the provision of evidence and data can increase accountability and build trust among water diplomacy actors…you guys are giving us hourly data updates once a day on your river gauges. It's publicly available information, but we need it twice a day. So [Country X] changed its behaviour and is now releasing its river gauge data, hourly data, twice a day, once every 12 h. And that's helped us also provide more timely warnings to the downstream also. So, that's Transboundary Impact or Transboundary outcome. I go and look at the reservoir and say what's the current level and if it looks to be high then I send them a note saying hey you know this is the time to watch, but that can be that all can be incorporated into a workflow that all can be automated and scaled up
P-11	[I]n the hard cases, it's often not the actual information[, and] that's the problem, it's the trust around between the parties. So, they don't trust each other and may not trust the data, may not trust the intentions that they think might be hidden in some in some data point, but they haven't been able to find out. So, and I think that part is one that's really hard where I don't really see how this can be easily addressed through AI because I mean it's a black box too…It's really hard to say and I think in the fundamental sense, I'm sceptical because if indeed the issue is one of trust, then I don't see how AI strengthened this trust
S-2	All is about data. While the major issues related to transboundary water raise the issue of data… with all these tools available like super computing power, we'll be able to have high-quality data…and these predictive AI models, we can predict much better when there's the accuracy. In terms of potential influence, the impacts of extreme events like and shifting water flows and availabilities can be better understood
S-3	[E]arly warning systems can be greatly improved with AI, enabling real-time monitoring and better assessment of the physical changes in a system and institutional capacity…If we can teach a machine to do what people do by hand, we can speed up the process and move into faster updates and ideally again into early warning where are the tensions going to be…Once upon a time, India or Israel or China could consider water data as a state secret, those days are gone. Now everybody has access to satellite information. So, you lose the information advantage of your hegemony and presumably now with AI that should democratize it even more. Now it's not just governments with access to the information, but anybody with access to Internet

**Table 3:** Deepening politicization due to distorted information and knowledge

Risks of malicious use of AI and knowledge politicization	
P-1	The fear of deepfake technology and its potential impact on trust and reliability is a concern…Digital meetings have become more frequent due to the pandemic but may face challenges if AI becomes more advanced in creating realistic avatars
P-4	I worry about AI's role in mis-and disinformation in general…Using artificial intelligence tools, that allows anybody to pretend that they're a lawyer, pretend that they're a diplomat, pretend that they're able to go out and negotiate, that's kind of dangerous because it will make people feel empowered, that they can do things, that there are other skills involved that they don't have…And then of course, I worry about mis- and disinformation and the ability to fake things. With AI obviously it is going to increase. And a lot of times as diplomats, we're trying to counter that mythology and bring people down to more of a fact based or evidence-based viewpoint of what's going on in the basin. And I suspect that artificial intelligence will expand our ability to strengthen those myths to our advantage. And so, I think that that creates a real problem…And I do think there's a huge issue around becoming intellectual hazards with the support of something like artificial intelligence
P-7	[A]nd if for example, now we know that there are tools that you can very easily record someone, you can already do it. But if you can, for example, disguise something or and then you can misinterpret it, you know you can use it for deepfakes…That can really affect people's willingness to be part of these kind of processes… it's a lot about trust and we don't want to have a situation when we will search people and ask them to hand in their phones and other devices. But I think these tools can be easily abused and if you for example want to their parties that would benefit from the railing process
P-9	AI amplifies disinformation campaigns, lowering the cost and enabling new actors. It spreads faster, impacting conflict narratives. … Deepfakes could undermine peace efforts, and sentiment analysis can tailor messages for specific audiences…Deepfakes could undermine trust, especially in areas with a history of conflict. The amplification and speed of disinformation campaigns through AI may erode truth further…It tailors content to specific audiences, potentially influencing support for policies. While not malicious, it raises concerns about the erosion of truth and the value of truth
P-11	[I]t isn't actually about the information, but about the trust, and the political narratives that are attached to…water conflict problems are much harder to resolve, because anything can be harnessed for disinformation jeopardising the process
S-1	If AI now, it can be used to generate what can be called fake news or things that look plausible, both in terms of scientific studies or in terms of news videos. You can have photos of the Ethiopian dam, which is completely filled. And so then the question is how those images, those stories, those narrative are used and by whom. So of course I think being more and more intelligent and powerful, those technologies and be used, I mean can empower people. But in the end for me is the is the goal and how people are using it for which purpose that remain what we can what we should scrutinize
S-3	[T]here is no controlling this [malicious use]…both the promise and the peril is once everybody has access to everything, and everybody can manipulate everything, if everybody can fudge data or put out false information…I think the genie's out of the bottle and we just have to do our best to hope that the that the like every technology that the forces of good prevail…if you want to sabotage an agreement, can you also be monitoring social media and couch something in precisely a way that would inflame tensions between the two sides?
S-5	[T]hey [AI systems] are of course prone to manipulation, and I'm not sure if we should base decisions and negotiations on which so many people then depend for, for example, drinking water or food security on something that a computer-generated [report] that we don't understand anymore
Unequal AI capabilities to reinforce knowledge politicization	
P-2	[T]he exclusion of the for example, parties or stakeholders that are not technologically advanced. If we say if there is a water diplomacy between Germany and the Netherland over the Rhine river, then we can imagine that both countries are equal in terms of technological adoption. But if we compare the water diplomacy between the Central Asian states or Pakistan, Afghanistan or Pakistan, India, then there are challenges of data tempering, data manipulation, exclusion of one group and inclusion of another group because India is technologically stronger than Pakistan and then Pakistan is technologically stronger than Afghanistan
P-2	[Country A] don’t have the capacity. [Country B] is a big country that has the institutional capacity, and they can manipulate the data…That’s why [Country A] refuses the system. [Country A] doesn’t need it
P-4	[A]gain, those countries that are already ahead, they're going to more rapidly be able to utilize artificial intelligence to advance their own interests and will that then exacerbate some of the inequalities that we see between within these negotiations over some of these water issues…And whoever controls the pen controls the, you know, controls the conversation
P-7	It also means who has access to these technologies. In certain basins, one country might have much more capacity to absorb these new challenges. So, it's even widening the gap. I think that here, our ability to, for example, understand how much water we have now and how much water we will have in the future, that's the due to new technology
P-11	[M]y assumption would be that the AI capabilities would be also fairly unequal in terms of how easily people can work with this and how easily they can perhaps manipulate or be perceived to manipulate that it will reinforce some of the sort of unhelpful dynamics…Well, the weaker countries didn't trust the data which was stored in the in the one of the stronger countries because that was where it had always been and where the expertise were
S-5	Because I think in a way as I said, they [AI] can leverage the playing field a bit. But I think they also come with a lot of risks because it might still be the more powerful in terms of technical and financial capacity countries that could use them…So, for [example programme], that's all open. I mean it's paid for by [financial donor] and they use it, but it's also open and anyone can use it. And even it's not only the results that are open but also the entire coding behind it is all available and the other countries don't make it available. And how many countries are there that can develop something like this in the first place at all? It's maybe 10, 15 countries around the world. So, what about the others? And what if a country that can do this would be negotiating over water with a country that can't? It's basically like this old debate about inequality over data…I think that's just next level now, same problem at much, much bigger level

**Table 4:** AI's potential for social learning and consensus building

Interview	Quotation
P-7	[I]n some basins, for example, the negotiating language is English, even though it's not the language of the region…And we know now that you can use AI or translations…And so, this could immensely help different negotiators. We see it often that some countries who negotiate, they actually have to rely on English and neither side is very strong in English. And you can also have a lot of misunderstandings…So maybe it's AI if you can use it as a tool to improve language understanding that can also help different processes to move forward…And it's sad that some people don't have ability to fully contribute to the negotiations because they just have hard time expressing themselves…But I think the technology that is out there, we can see it in the future
S-2	AI might also aid in communication, facilitating multi-stakeholder dialogues through translation tools
S-3	Water diplomacy involves both water-related data and the art of negotiation. AI can contribute not only to information but also to strategies and problem-solving, potentially facilitating collaborative governance…As technology progresses, the information advantage of hydro-hegemons diminishes. Initially, those with access to data or satellite information had an upper hand, but with AI and widespread internet access, that advantage diminishes further, potentially equalizing negotiating positions…transparency and access to information will help balance power dynamics

**Table 5:** AI bias and governance risks

P-1	The transparency of AI tools is crucial for gaining trust among users…Convincing people to use AI will be challenging if it's considered a black box
P-2	So my indigenous knowledge is much more effective than the artificial intelligence. And if artificial intelligence replaces my indigenous knowledge, then…but there won't be any emotions, any feelings. So, in that case you need emotions, you need feelings to allocate more water so that you can actually remove the poverty…India is a big country that have the institutional capacity and they can manipulate the data…That's why we refuse this system
P-3	[H]owever, trust is paramount. Technology developers must recognise their role as partners, ensuring transparency and adapting to human conditions…Transparency is key, irrespective of the developers. Trust is built through an open approach. The EU's data access initiative highlights the importance of data for individuals globally. Developers must consider trust, transparency, and adjustability to human conditions…Diplomats need to be open minded, and technology developers must start from scratch, building trust through transparent processes. The aim is not a solution but a partnership in navigating the complexity of global challenges. Trust is a gradual process that technology must respect
P-4	[T]here are governments that would create a doomsday scenario within their own AI systems so that even if they are no longer capable of responding or controlling, those systems would take over and execute whatever functions that they wanted to have happen including their water sector
P-5	So, if you have to move to that new West technology, I think we still cannot abandon these traditional technologies yet, because this has already been built for so many years
P-8	[A]nd my point was, but probably we should not expect completely new decisions in in diplomacy practice or insights that the artificial intelligence can suggest. So, it's not cancelling the human creativity and we should not expect that they will be providing all solutions for everything. It's just some tools that we can use to you know to substitute the lack of information or experience from somewhere else and to build on this our own practice and experience
P-11	[I]n the hard cases, it's often not the actual information[, and] that's the problem, it's the trust around between the parties. So, they don't trust each other and may not trust the data, may not trust the intentions that they think might be hidden in some in some data point, but they haven't been able to find out. So, and I think that part is one that's really hard where I don't really see how this can be easily addressed through AI because I mean it's a black box too…It's really hard to say and I think in the fundamental sense, I'm sceptical because if indeed the issue is one of trust, then I don't see how AI strengthened this trust
S-1	We as a human and we are still in charge and perhaps better equipped than artificial intelligence, I think. But of course, we are also using information knowledge generated through artificial intelligence and to manage and decide or trying to influence those relations
S-2	It is the effect but then we don't really know what to trust…there are so many different AI models, and we really have no idea what AI is capable of
S-5	[H]ow do we know that this is the best decision? And if it wasn't, who takes responsibility for it later? kind of also releases decision makers from responsibility in a way…I think yes, of course it's always the decision maker that is responsible in the end…And then this completely fails because it was not a good strategy and effects people's access to water. The decision maker can always say, oh, but AI told me to do this, I'm out, you know… And if we feed [data] into AI, and AI identifies patterns… by looking for the strongest patterns you can get this kind of reinforcement. And then maybe the recommendation that comes out of let's say a negotiation modelling could be a more conflictive option than if a human had chosen it. So, I see it as a risk

## Results

In this section, expert insights are analyzed for examining theorized propositions. We focus on AI’s implications to objective knowledge production, politicizing knowledge, and knowledge for consensus building in international water dispute management.

### Technical strides to objective and credible data and information

The expert interviews emphasize the potential benefits of integrating AI technologies for new data that can facilitate water cooperation and negotiations. As we discussed earlier, water- and diplomacy-related data and information can be limited in accuracy and credibility (Fröhlich 2012; ter Horst et al. 2023b). Emerging narratives stress an opportunity that AI’s superior computational power to drastically enhance the measurement, speed, and accuracy of data production and promote information transparency.

#### Hydrological forecast and transboundary early warning powered by AI

Data collection and exchange is a crucial element for water diplomacy as efforts to prevent, mitigate, and resolve water-related disputes, as one scholar puts it: “All is about data” (S-2, Table 2). Interviewed experts expressed their hopes that AI can be used to unpack future uncertainties of climate change and impacts of water infrastructure and environmental degradations, which have emerged as a more prominent concern in water negotiations and transboundary water governance arrangements (Rivera-Torres and Gerlak 2021). In particular, machine learning techniques, using continuous input data to build a system that can carry out complex tasks and improve its performance over time, are frequently mentioned in describing the opportunities for generating better hydrological data. Two main outcomes mentioned in ‘new’ knowledge produced by AI-assisted systems are forecasting water trends and transboundary early warning of disasters. Eventually how these tools can inform negotiators and transboundary water managers about evidence regarding water use and distribution disputes is still to be observed.

Predictive modeling techniques and forecasting have been mentioned by several interviewees as a potentially positive contribution that machine learning-powered tools and models can make in mapping future water resources for enabling water agreements and other forms of cooperation (Table 2, P-2, P-4, P-5, P-6, S-2). Machine learning models can make predictions for hydrology and environmental and socio-economic conditions based on historical data. The ability to forecast trends is an asset in negotiation settings, because it can reduce uncertainty about the future and inform negotiators about their positions. Transboundary flood risk assessments by the Mekong River Commission and flood warning on the downstream Red River in Vietnam have recently advanced due to machine learning-powered predictions (Kittikhoun 2025; Motta and Koehler 2025, 7). These hazard warning tools may contribute to reducing disaster risks, but these benefits are not yet directly contributing to reducing tensions among water-sharing countries (International Crisis Group 2024). Negotiators can be more confident when they are informed about future water needs, which are better estimated with more precise, up-to-date, and accurate data (Table 2, P-2, S-2). Future water availability and variability have been a source of tension and barriers in negotiations and adjusting an existing transboundary water agreement (Rivera-Torres and Gerlak 2021; Shahbazbegian and Dinar 2023). Therefore reasonable forecast reports can reduce uncertainty and boost confidence in commitments for further cooperation and agreements.

AI tools can aid to enhance existing transboundary early warning and monitoring systems, enabling faster updates. One expert stated, “[I]f we can teach a machine to do what people do by hand, we can speed up the process and move into faster updates and ideally again into early warning where are the tensions going to be…” (Table 2, S-3). Amid climate change exacerbating the frequency and intensity of hazards and water scarcity, transboundary water risk management is a key concern of countries relying on shared waters, and it is increasingly becoming an important area of cooperation (Kåresdotter et al. 2025). When the frequency and quality of data one riparian country receives from another riparian country is insufficient for generating transboundary early warning reports, AI-assisted systems can fill the gap (Table 2, S-3). Such contribution of data can reduce international tensions around water use and allocation. How enhanced remote sensing capacity due to AI can foster transparency and cooperation is detailed in the subsequent section.

#### AI for data transparency and water cooperation

Water data sharing is not universal and absent in many transboundary contexts. Even if data are shared, the frequency and quality of data may not be sufficient for other water-sharing countries to have a shared understanding of the basin (Gerlak et al. 2011). The absence of credible and mutually accepted data is a problem to determine equitable and reasonable use and significant harms in transboundary water settings (Table 2, P-10). Transparency in water data is, in this sense, a crucial for addressing concerns and accountability in water governance. One practitioner explained, “the provision of evidence and data can increase accountability and build trust among water diplomacy actors” (Table 2, P-10). When parties have difficulties to establish mutually agreed information, remote-sensing tools provide credible data. Satellite image monitoring tools can detect changes in river flow, for example, and they help understand how human interventions like dams impact transboundary rivers (Stimson Center 2024).

AI, more specifically machine learning techniques, has a critical contribution to enhance the speed of satellite imagery processing by enabling automatic sorting and data curation. And this can promote openness in highly secretive water-related data (Hasan et al. 2023; ter Horst et al. 2023a). The advances made in open-access satellite imagery and radar technology are the enabler for more transparency and accountability in the water sector including the monitoring of invisible groundwater (Collignon 2020; Kamali Maskooni et al. 2020). Automated data collection and curation can enable near real-time tracking of impacts, which can facilitate the timely identification of hydrological patterns and hazards, thereby improving strategies for addressing both climate-related and infrastructure-related water risks (Table 2, P-10, S-3). Automated monitoring and real-time analysis can contribute to a timely workflow, paving the way for scale-up of monitoring processes (Table 2, P-10).

To this day, no case has been reported on water-sharing countries to deploy an AI-enabled remote sensing system for joint data collection for transboundary water management. To what extent such use of AI is possible or desired is still to be surveyed, but some countries and actors have more appetite for such use than others. Despite such uncertainty, some interviewed experts shared an optimistic view that advanced monitoring tools can help establish mechanisms that promote accountability and transparency in transboundary water governance (Table 2, P-9, S-3). Facilitating discussions on AI-powered monitoring tools may become part of water diplomacy efforts in the future.

The interviews have emphasized their hopes for automation by AI for expediting, scaling up, and streamlining water diplomacy processes. For instance, the automated communication between governments and water diplomacy actors can speed up the negotiation processes. In addition, water diplomacy information such as diplomatic protocols, relevant national, and international laws, and socio-hydrological data can feed into training an AI system that can provide timely inputs during negotiations. Such systems can enhance the negotiators’ ability to identify opportunities for further collaboration during water diplomacy processes (Table 2, P-4). AI tools can be used to detect certain text and images in screening and verification processes during water negotiations (Table 2, P-9). AI has the potential to enhance resource and time efficiency and to inform decision-making in both long-term and short-term (Table 2, P-4, P-9, P-10). This acceleration can enable water authorities, diplomats, and policymakers to respond more swiftly to emerging water-related challenges, such as transboundary flood and drought risks (Table 2, P-2, P-5, P-10, S-3).

Despite the technological advances, AI systems are prone to manipulation and bias (Debnath et al. 2023). Concerns about the impartiality of water data have been a perennial issue in water dispute management, and experts were not in agreement on how the use of AI will influence trust on data (Table 2, P-6). Some mentioned that AI-assisted models and data collected through AI can only be trusted if a neutral and credible third party is responsible (S-3), while others raised the fundamental problems of impartiality and biases in models (S-5). Disparity in technical capacities among water-sharing countries amplifies such concerns about distrust (Table 3), leading to the refusal of certain knowledge platforms (Table 2, P-2). The lack of transparency in the development of AI tools for water management poses a risk of deepening mistrust among water-sharing countries, if one country proposes to develop such a tool. In the following section, how AI would exacerbate politicization of information by producing and spreading distorted outputs is outlined.

### Deepening politicization due to distorted information and knowledge

Water has been politicized through the deliberate production of certain knowledge, with parties able to determine what is researched or left unknown (Zeitoun et al. 2011). There is political influence over data generation, information sharing, and knowledge production, but the emergence of AI exacerbates the concerns about contested use of data and information. Manipulated and biased water data has long been a point of tension in water diplomacy, because riparian countries inevitably fear flawed data to be used against them. Such fear undermines the trust of water-sharing countries and complicates political processes. Experts expressed their concerns about AI to exacerbate the risk by being used to spread misinformation and disinformation about water usage and its impacts among neighboring countries, further politicizing water. The following section outlines the results related to AI’s role in amplifying the politicization of water disputes through knowledge manipulation and contestation.

#### AI to produce mis- and disinformation about water

Systematic (re)production of false information by using generative AI is a serious concern (Future of Life Institute 2024). In the context of water diplomacy, the manipulated or false information about water scarcity and water infrastructure has unraveled trust and relationships (Klimes et al. 2023). The risk of spreading misinformation can be done unknowingly. For instance, individuals who lack the necessary expertise can claim to be experts using open-access AI tools or present AI-generated content as factual knowledge without proper verification (Table 3, P-4). Such risks of skipping verification on AI-generated content similarly apply to the use of AI tools by water diplomacy actors.

The misuse of AI-generated fake news, scientific studies, and manipulated images or videos related to water disputes is a significant concern mentioned by several experts (Table 3, P-1, P-7, P-9). Deepfakes, which combine deep learning with fake content, are “hyper-realistic videos digitally manipulated to depict people saying and doing things that never actually happened” and “rely on neural networks that analyze large sets of data samples to learn to mimic a person's facial expressions, mannerisms, voice, and inflections” (Westerlund 2019, 39). Political actors can strategically produce deepfakes, and their involvement in escalating tension over water is concerning (Klimes et al. 2023). This technology could be employed to fabricate evidence or narratives to serve specific agendas, undermining trust and reliability in information related to water management (Table 3, P-1, P-4, P-7, P-9, P-11, S-3, S-5).

While disinformation campaigns are not new, the rise of generative AI is expected to amplify and accelerate such campaigns, especially in regions with a history of conflict (Table 3, P-9). The regulation of malicious AI use in water dispute management remains unclear, though mitigation measures such as physical meetings and rigorous screening tools are available for verification (Table 3, P-1, P-9). However, a lack of effective oversight and accountability mechanisms poses questions about the impact of spread of misinformation and the erosion of trust in diplomatic processes. Contestation over the truthfulness of knowledge may deepen further in the AI era.

Digital technologies and platforms can also become spaces where individuals express strong emotions regarding transboundary water concerns (Deka et al. 2023; Seide and Fantini 2023). AI-generated images and information can be used to spark emotions, as generative AI tools have already influenced public opinion on sensitive issues (Kreps and Kriner 2023), The risk of AI’s malicious use is difficult to mitigate when parties have varying degrees of technical capacities to verify such information. The following section elaborates how unequal AI capabilities further exacerbate politicization of water disputes.

#### Unequal AI capabilities amplify inequality among conflicting parties

Uneven technological capacities between parties contribute to power imbalances, and AI is likely to deepen power asymmetry in water disputes. The availability and affordability of advanced AI technologies, such as radar and satellite imagery and machine learning algorithms, vary significantly among nations (Galaz et al. 2021). The disparity in AI capacity can amplify existing inequality among water-sharing countries (Table 3). Currently, the capacity for hydrological modeling and infrastructure development significantly vary among countries in major transboundary river basins such as the Nile and the Mekong (Gao et al. 2022; Hayat et al. 2022; Table 3, P-4, P-11). Developing AI systems would require substantial financial resources and technical capabilities that are unevenly distributed among water-sharing countries (Table 3, P-10). Countries with more advanced technical capabilities are better positioned in the negotiations as hegemons, and the power dynamics influence the interactions over shared waters (Zeitoun et al. 2017). When a technologically advanced party can gather data remotely without the consent of a weaker counterpart, the technically powerful party can effectively undermine the latter's ability to resist the data collection (Zeitoun et al. 2011). The more unilateral data collection is done by using AI tools, less trust and consensus building is likely among water-sharing countries (Table 3, S-5). Moreover, the ability to manipulate data or disseminate misinformation through AI-powered tools raises concerns about the escalation of tensions and mistrust among riparian states (Table 3, P-2, P-2, P-11). Such strategic interventions can hinder knowledge to be further shared for the common good, complicating cooperation over transboundary water.

Not all AI-assisted data collection aims to manipulate data and knowledge to serve self-interest of powerful countries, but this is a legitimate concern (Table 3, S-5). The countries with more technical capacities are more likely to adopt AI technologies and thus benefit, leading to “even widening the gap” in negotiations (Table 3, P-4, P-7, S-5). Particularly those conflict-affected and fragile countries will face additional challenges in developing capacities and accessing AI technologies (Table 3, P-7).

AI can amplify existing power asymmetries in transboundary water interactions and deepen the politicization of knowledge and disputes (Mirumachi 2020). A tool is only as good as the person who uses it, and AI is also a tool that can be used for either contestation or collaboration. Some experts expressed that AI can be used for consensus building in water diplomacy by serving as a platform for cooperation and addressing capacity inequalities, as is discussed in the following section examining the third proposition.

### Consensus building through social learning and co-production

Water diplomacy’s ultimate aims include preventing, mitigating and resolving water disputes, and seeking consensus building involves a process encouraging good-faith engagement and seek a unanimous agreement. Experts highlighted a potential equalizing effect of open-source AI tools in uneven playing fields, mitigating power imbalances and promoting equitable collaboration among actors involved in water dispute resolution (Table 4, P-7, S-2). Open-source AI systems that are freely available to use, study, modify, and share can inform countries with weak institutional knowledge and hydrological information during negotiations. Having stated the risk of ‘misinformation’ generated by AI in 4.2, such use of AI should be cautioned with a degree of verification efforts by individual users (Maleki et al. 2024). If used adequately, open-source AI systems can provide timely information, which is particularly useful to countries with less resources and weaker technical capacity. This leveling field effect can encourage more meaningful participation in negotiations and reassure their commitments.

Open-source AI can also facilitate capacity building for strengthening water diplomats’ competence, especially authorities from fragile- and conflict-affected states as well as countries with limited resources (Table 4, P-7). For instance, AI-powered translation tools can serve as a communication aid that enables broader participation and collaboration in diplomatic negotiation (Table 4, P-7, S-2). The language barrier is a disadvantage to parties with insufficient human and technical capacities by inhibiting them from actively participating in informal talks and building relationships with their counterparts (Table 4, P-7). The use of natural language processing model for simultaneous translation may fill this gap in the future, while state-of-the-art machine translation is yet to be perfected for diplomatic settings.

If government officials, negotiators, and civil society actors can equally benefit from open-access AI tools, technology can contribute to strengthening the capacity of technologically disadvantaged actors. A range of affordable AI subscription services is now available to support various capacities including research, text and image generation and editing, and analysis. These AI generated contents may not be as credible and relevant as reports produced by human researchers and analysts, but, at least, policy makers can quickly gain basic information and overview. This type of AI use has a potentially equalizing effect in negotiations by bringing balance of power to a degree (Table 4, S-3). AI tools can enhance processing and analysis of the remote sensing data (see the discussion in 4.2), and this additional information may reduce the information disparity and foster cooperation (Table 4, S-3).

AI and related technologies can lower the barriers to participate in water diplomacy processes. Active participation and meaningful engagement can promote collaborative learning, which is essential for producing a shared understanding and knowledge and seeking solutions for transboundary water disputes (Sadoff and Grey 2002). The exact prospects for such use of AI still need to be explored and tested, as experts suggest, but there is certainly a possibility to enhance social learning aspects in water diplomacy by using AI.

## Discussion

The evaluation of three propositions of AI’s implications to international water disputes reveals a degree of uncertainties, but also several underpinning dynamics that could influence the potential impacts of this emerging technology. First, there is limited evidence that AI tools generate more neutral and credible data, or that the new knowledge they produce facilitates greater cooperation among water-sharing parties. While various pilot projects and exploratory research have tested the use of machine learning in hydrological modeling, risk assessment, and monitoring systems, the results remain inconclusive. For example, transboundary flood warning systems in the Mekong and Red rivers demonstrate some of the clear benefits of AI’s forecasting capabilities. However, how responsible governments and authorities act upon these new warnings remains unclear. So far, no water-sharing parties have attempted to initiate new negotiations based on this knowledge.

Then, experts have raised cautions toward the increased tensions over transboundary waters due to the malicious use of AI to generate disinformation that can politicize and emotionalize water disputes, and unequal power distributions would worsen this risk (also see Klimes et al. 2023). The second perspective of AI to exacerbate politicization has the most spelled out and concrete examples and concerns, based on experts’ experience and knowledge of the evolving field. A series of research works on the dam dispute in the Nile River basin illustrate how the social media campaigns and public emotions form critical inputs in water negotiations, which suggests the risk of AI-generated mis-and disinformation on a politically sensitive context.

Technological uncertainties innate to AI such as its ‘black box’-like nature and algorithmic opaqueness undermine the quest for unbiased data and transparency in water governance and negotiations (Rudin 2019). The black box like nature of AI is a general problem in understanding the outputs generated by AI systems for policy relevance (Table 5, P-1, S-5). This transparency issue is also closely linked to the accountability question of using knowledge generated through AI tools for high-stake policy making. The stakes are high if the accuracy of AI-generated intelligence determines how much water should be available for a country, a region, or a community (Table 5, S-5). The question on accountability for decisions made based on AI-generated intelligence is critical, as the involvement of AI can release decision-makers from responsibility (Table 5, S-5). Ultimately, the use and purpose of AI-generated knowledge is a political responsibility (Table 5, S-1).

There is a call for improving AI data pipelines, because how data are collected, curated, and used to build AI systems fundamentally determine the quality of AI systems that are built as results (Liang et al. 2022). The truthfulness of data and information may become even more contested in the future because of the increasing fear of data manipulation and hidden bias in AI systems (Qamar et al. 2019). This seems to be closely linked with the expressed distrust and concerns, whether policymakers should consider intelligence from such systems (Table 5, P-1, P-11). These issues have been addressed in regards to socio-economic, security, public health and sustainability impacts of AI (Galaz and Mouazen 2017; Francisco and Linnér 2023; Kim and Boulanin 2023). What an algorithmic transparency and explainability would influence water negotiations and dispute management between countries needs further research (see Leichtmann et al. 2023 for various efforts in making AI models and outcomes more explainable). The question on how AI-assisted tools can overcome distrust and biases remains critical to achieving mutually accepted knowledge contributing to peaceful management of shared waters.

Timely discussions on the governance of AI in water diplomacy are warranted since there is a notion of *inevitability* of AI. Several experts mentioned that they would eventually *need* to use AI tools (Table 5, P-5, P-8). This indicates a degree of the passive acceptance of AI among practitioners working on water diplomacy, while various industries and public sectors actively adopt AI tools (Kelly et al. 2023). Several respondents provided examples of how AI is used in data collection and improving language in policy deliberations (Table 5, P-1, P-7, S-1). The absence of internationally agreed guidelines and policies on the development and use of AI systems in water diplomacy may pose a risk in the future.

The need for institutional arrangements to govern AI tools in water dispute management is further emphasized by the profound concerns by experts regarding AI-generated disinformation and disruption. Global AI governance is still emerging, and international water management and governance practitioners and scholars have not engaged in discussing necessary guidelines and principles in the application of AI in this field. Despite the alarming risks, AI technologies have been slowly applied in knowledge production, informing controversial water dispute discourses such as those in the Nile Basin (ter Horst et al. 2023b). There is a growing need to closely observe how these technologies are perceived by negotiators and utilized by various actors in the complex chains of water diplomacy, which future research can further explore. This negative side of AI in water diplomacy may hinder conflict parties as well as relevant third parties from exploring the use of AI in various activities.

Finally, lessons from existing initiatives supporting consensus building and social learning in transboundary water governance highlight potential entry points for open-source AI tools. Parties can be reluctant or even reject new data if they resist the power asymmetry embedded in knowledge production (Table 5, P-2, S-5). Enhancements in data and analytics alone cannot solve complex and long-running water disputes. In fact, advanced technologies and tools may worsen tensions due to perceived biases (Table 5, P-8). In addition, the general risk of AI systems can lead to unknown risks, especially since these systems are developed without sufficient human supervision and consideration for human emotions (Table 5, P-2). These developments can undermine universal values and morality in water allocation, which are developed through decades of interactions that result in norms (Grech-Madin et al. 2018).

## Conclusion

The article focuses on the intersection of AI and water diplomacy by examining the expert narratives on the risks and opportunities. As our findings suggest, positive impacts of AI technologies are still to be seen in international water dispute management efforts including water diplomacy. These future AI tools need to more actively acknowledge the political nature of knowledge production and the unavoidable political undertones within seemingly purely technical efforts (Wheeler et al. 2023). With the rise of AI, technical capacities may have an even greater impact in transboundary water governance (Mirumachi 2020; Vij et al. 2020; Döring et al. 2024). How such impact would influence sustainability and peace linked to water is a further question that should be answered through empirical studies.

The described risks related to knowledge production and the politicization of knowledge suggest that care must be taken when incorporating AI into international water dispute management. Offering training for water conflict mediators and water management authorities is one way to strengthen their capacity to understand and potentially utilize these tools peacefully. If such activities are carefully designed and involve well-chosen participants, they can help reduce disparities in technical capacity among water-sharing parties. The findings also emphasize the importance of maintaining human control in water diplomacy processes. Physical, in-person meetings and human interactions remain indispensable for resolving international water disputes, even when relevant data can be accessed remotely through sensing technologies. Additional training and capacity building in science communication are increasingly necessary to ensure these AI tools are properly understood when deployed. Furthermore, future research should contribute to the development of clear guidelines for the responsible design and use of AI systems in water diplomacy. This effort should be part of a broader, collaborative process that brings together diverse stakeholders to ensure AI tools support equitable and informed decision-making in transboundary water governance.

## Supplementary Information

Below is the link to the electronic supplementary material.

Supplementary material 1 (PDF 56 kb)

## Data Availability

Data used in this research is confidential and not made available. All interviews were conducted under conditions of anonymity, and participants did not provide consent for the publication of interview transcripts. Given the sensitive nature of water diplomacy and the potential for identifying participants through their responses, making the data publicly available would compromise participant confidentiality and violate the ethical terms under which the research was conducted.
