# Supplementary material for: Making water knowledge with Artificial Intelligence: A qualitative study of expert interviews on water diplomacy
Source: Ambio. 2025 Nov 14;55(4):857–74. doi: 10.1007/s13280-025-02272-z (PMC12961011; doi:10.1007/s13280-025-02272-z)
Supplement: Supplementary file 1 — Supplementary material 1 (PDF 56 kb) [file 13280_2025_2272_MOESM1_ESM.pdf]

Ambio

Supplementary Information

This supplementary information has not been peer reviewed.

Title: Making water knowledge with Artificial Intelligence: A qualitative study of expert interviews on water diplomacy

<u>Interview questions</u>

General Questions

1. How familiar are you with water diplomacy? Would you consider yourself an expert, practicioner, or policymaker in this field? Multiple choices are possible.
2. In your opinion, what are the key challenges in current water diplomacy efforts?

Artificial Intelligence (AI)'s Role in Water Diplomacy:

3. How familiar are you with digital tools including AI?
4. Can you provide existing examples of how AI has already been applied?
5. Do you envision AI as a tool for water diplomacy? In other words, can AI facilitate cooperation among countries and actors in managing shared water resources?
    a. If yes, please explain how.
    b. If not, please explain why.
6. How do you foresee the role of AI evolving in the context of water diplomacy over the next decade?

Risks and Concerns:

7. What are the potential risks and challenges associated with using AI in water diplomacy? Are there any ethical, technical, or political concerns to consider?
8. How might the use of AI in water diplomacy impact human decision-making and the role of human mediators or negotiators?
9. What data privacy and security considerations should be taken into account?

Capacity building and cooperation

10. What skill sets would be essential for mediators and diplomats to effectively work alongside AI tools in water diplomacy processes in the future?
11. Are there lessons from other domains, such as environmental conservation or conflict management, that could inform the responsible and effective integration of AI in water diplomacy?

Closing...

12. Any other reflections on the issues related to AI and water diplomacy?
